# Preoperative prediction of Lauren classification in gastric cancer: a radiomics model based on dual-energy CT iodine map

**DOI:** 10.1186/s13244-023-01477-8

**Published:** 2023-07-16

**Authors:** Min Li, Hongtao Qin, Xianbo Yu, Junyi Sun, Xiaosheng Xu, Yang You, Chongfei Ma, Li Yang

**Affiliations:** 1 Department of Computed Tomography and Magnetic Resonance, Fourth Hospital of Hebei Medical University, No. 12, JianKang Road, Shijiazhuang, 050010 Hebei Province People’s Republic of China; 2 Department of Radiology and Nuclear Medicine, The First Hospital of Hebei Medical University, No. 89, Donggang Road, Shijiazhuang, 050031 Hebei Province People’s Republic of China; 3 Siemens Healthineers Ltd., 7, Wangjing Zhonghuan Nanlu, Beijing, 100102 People’s Republic of China

**Keywords:** Gastric cancer, Lauren classification, Dual-energy CT, Iodine map, Radiomics

## Abstract

**Objective:**

To investigate the value of a radiomics model based on dual-energy computed tomography (DECT) venous-phase iodine map (IM) and 120 kVp equivalent mixed images (MIX) in predicting the Lauren classification of gastric cancer.

**Methods:**

A retrospective analysis of 240 patients undergoing preoperative DECT and postoperative pathologically confirmed gastric cancer was done. Training sets (*n* = 168) and testing sets (*n* = 72) were randomly assigned with a ratio of 7:3. Patients are divided into intestinal and non-intestinal groups. Traditional features were analyzed by two radiologists, using logistic regression to determine independent predictors for building clinical models. Using the Radiomics software, radiomics features were extracted from the IM and MIX images. ICC and Boruta algorithm were used for dimensionality reduction, and a random forest algorithm was applied to construct the radiomics model. ROC and DCA were used to evaluate the model performance.

**Results:**

Gender and maximum tumor thickness were independent predictors of Lauren classification and were used to build a clinical model. Separately establish IM-radiomics (R-IM), mixed radiomics (R-MIX), and combined IM + MIX image radiomics (R-COMB) models. In the training set, each radiomics model performed better than the clinical model, and the R-COMB model showed the best prediction performance (AUC: 0.855). In the testing set also, the R-COMB model had better prediction performance than the clinical model (AUC: 0.802).

**Conclusion:**

The R-COMB radiomics model based on DECT-IM and 120 kVp equivalent MIX images can effectively be used for preoperative noninvasive prediction of the Lauren classification of gastric cancer.

**Critical relevance statement:**

The radiomics model based on dual-energy CT can be used for Lauren classification prediction of preoperative gastric cancer and help clinicians formulate individualized treatment plans and assess prognosis.

**Graphical abstract:**

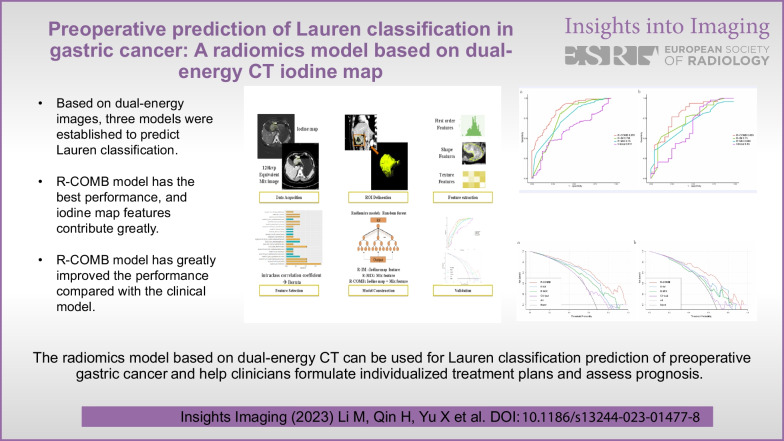

**Supplementary Information:**

The online version contains supplementary material available at 10.1186/s13244-023-01477-8.

## Key points

1. Based on dual-energy images, three models were established to predict Lauren classification.
2. R-COMB model has the best performance, and iodine map features contribute greatly.
3. R-COMB model has greatly improved the performance compared with the clinical model.

**Supplementary Information:**

The online version contains supplementary material available at 10.1186/s13244-023-01477-8.

## Introduction

Gastric cancer is one of the most common cancers worldwide and the third leading cause of cancer-related deaths [1]. Lauren classification is a common histological classification method that categorizes gastric cancer into intestinal, mixed, and diffuse types [2]. The intestinal and diffuse types have different clinical behavior and molecular features, and the clinicopathological manifestations and prognosis of the mixed type are similar to those of the diffuse type [3, 4], Lauren classification is an important indicator of prognosis for patients with gastric cancer [3, 5]. It has been shown that postoperative recurrence rate is higher in diffuse gastric cancer than intestinal type [3, 6], and 5-year overall survival rate is higher in intestinal gastric cancer than diffuse [3, 5]. Adjuvant chemoradiotherapy improves disease-free survival in patients with intestinal gastric cancer, but not in patients with diffuse gastric cancer after D2 resection [7]. In addition, patients showed different sensitivities to chemotherapy according to Lauren classification, with diffuse patients showing a higher efficiency to chemotherapy [8]. More importantly, in clinical practice, the surgical strategy can refer to Lauren classification [9], where diffuse gastric cancer is highly invasive and the extent of surgical resection is greater than that of intestinal gastric cancer, and these patients often require adjuvant chemotherapy after surgery [10, 11].

Postoperative histopathological examination is the gold standard for determining Lauren classification, but there is a lag in obtaining Lauren classification through postoperative pathology. Although Lauren classification can be obtained preoperatively by gastroscopic biopsy, it is not only invasive but also few tissue specimens, which has a significant impact on the diagnostic accuracy of Lauren classification [12]. The literature reports that the concordance rate of Lauren classification between biopsy and surgical samples is only 64.7% [13]. Therefore, accurate preoperative Lauren classification of gastric cancer can facilitate individualized treatment and improve prognosis.

Computed tomography (CT) is a convenient and fast examination option for patients suspected of having gastric cancer. Some studies have found that morphological features such as tumor size, location, and enhancement pattern in gastric cancer are correlated with the Lauren classification. However, due to a lack of quantitative parameters and diagnostic thresholds, the value of traditional imaging features in predicting the Lauren classification is limited. In this regard, dual-energy CT (DECT) is a novel imaging modality that brings CT-based diagnosis from the morphological to the functional field by employing iodine mapping (IM) to quantitatively reflect the lesion’s blood supply [14]. A few imaging studies have reported positively on the diagnostic value of iodine mapping in gastric cancer [15, 16].

Radiomics transform visual information from imaging data into a large number of deep digital features for quantitative studies. Through feature extraction and dimensionality reduction, high-dimensional features with great stability and reproducibility related to the disease's biological behavior can be used for model building, which allows improved objective quantitative assessment and has potential advantages in tumor precision assessment. The value of radiomic for the serosal invasion [17], evaluation of lymph node metastasis [18], prediction of occult peritoneal metastasis [19], treatment effect and prognosis prediction radiotherapy effect and prognosis [20, 21] in gastric cancer has been reported in previous studies.

Based on the existing knowledge of radiomics, we hypothesized that a radiomics model based on a DECT-IM may contain abundant quantitative parameters to determine the Lauren classification of gastric cancer more accurately. Accordingly, in this study, we attempted to establish and evaluate a preoperative radiomics model based on DECT venous-phase IM and 120 kVp equivalent mixed images (MIX) to predict the Lauren classification of gastric cancer.

## Materials and methods

### Characteristics of patients

This study was approved by the Ethics Committee of The Fourth Hospital of Hebei Medical University. We retrospectively reviewed the medical records of gastric cancer patients who underwent surgery between April 2015 and December 2017 at our institution. The following criteria were used for inclusion: (1) The patient had not received any anti-tumor treatment before surgery; (2) a dual-energy abdominal dual-phase enhancement CT scan was performed within 2 weeks preoperatively and the complete imaging data were available; and (3) postoperative pathology of gastric adenocarcinoma was conformed to clear Lauren classification. Patients were excluded in the case of (1) inadequate preparation before the CT examination, such as insufficient gastric filling or excessive gastric contents, which may affect the visualization of the lesion; (2) the presence of breathing or sclerotic artifacts in the image; and (3) the thickness is less than 0.5 cm (the range of the ROI is difficult to delineate).

### Imaging protocol and postprocessing

#### Pre-inspection preparation

All patients were made to fast for 6 h before the examination. Scopolamine (10 mg) was injected intramuscularly 10 min before the scan and 800–1000 mL water or 6 g aerogenic powder was given orally to fill the gastric cavity.

#### Examination method and scanning parameters

All CT scans were performed using a Siemens second-generation dual-source CT (SOMATOM Definition Flash; Siemens Healthcare, Germany) in the supine position. The scan was performed from 5 cm above the right diaphragm (at the level of the inferior pulmonary vein) to the superior border of the pubic symphysis. The following parameters were used during the plain CT scan: tube voltage: 120 kVp; tube current: 210 mAs; collimator width: 128 × 0.6 mm; collimator pitch: 0.9. Non-ionic contrast agent (Iohexol, 300 mg/dL; GE Healthcare, USA) was injected intravenously through the elbow median vein at a flow rate of 3 mL/s (2 mL/kg body weight). Two phase enhanced with dual-energy scans were performed at 25 s (for the arterial phase) and 70 s (for the venous phase) after injection. Enhancement scan parameters were as follows: tube voltages: A: 100 kVp, B: 140 kVp; Care Dose: 4D on, reference tube currents: A: 230 mAs and B: 178 mAs; collimator width: 32 × 0.6 mm; collimator pitch: 0.55.

### Image reconstruction and postprocessing

The dual energy data of the venous phase with a slice thickness of 1 mm were transferred to a workstation (SyngoMMWP, VE36A) and analyzed by applying the LiverVNC mode of the dual energy software to obtain IMs. The IM and 120 kVp equivalent MIX (weighted factor: 0.5) were used together to construct the radiomics model.

### Clinical model development

#### Traditional features

We collected the demographic data of the patients, including gender, age, and serum tumor marker levels (CEA, CA19-9, CA72-4).

*Analysis of image semantic features*: Two observers with 10 (L.M.) and 17 years (Y.L.) of experience in abdominal diagnostic imaging independently analyzed image semantic features using a dichotomous classification method by combining the axial and MPR images without knowing the pathological findings. Referring to the American Cancer Federation (AJCC) 8th Edition TNM staging system for gastric cancer, in the case of any disagreement, a consensus was reached through negotiation.

The image semantic features included:

- Tumor range: The stomach is divided into four parts: cardia, fundus, body, and antrum; tumors involving a single part are considered a single region, whereas those involving two or more are designated as multiple regions.
- Tumor location: Bounded by the middle of the stomach, the tumor was divided into the proximal stomach and distal stomach.
- Tumor thickness: The maximum tumor thickness is measured perpendicular to the stomach wall.
- Tumor enhancement form: The difference between the maximum CT value and the minimum CT attenuation value in the venous stage of cancer is considered uniform enhancement if the difference is < 10 HU, and inhomogeneous enhancement if the difference is ≥ 10 HU.
- Degree of tumor enhancement: CT attenuation of the venous stage carcinoma with a net added value of ≥ 40 HU was marked enhancement, while those with < 40 HU were not obviously enhanced.
- Clinical T (cT) staging: tumors with an unsmooth serosal surface, mural nodules, and fuzzy fat space were considered a T4 stage, and those with a smooth serosal surface were considered a non-T4 stage.
- Clinical N (cN) staging as cN (−) or cN (+) lymph nodes: Regional lymph nodes, round or ovoid, with a short diameter (≥ 1.0 cm) or clustered with enhanced small lymph nodes.

#### Screening traditional features and establishing clinical models

Based on the training set data, the traditional features related to the Lauren classification were analyzed by univariate analysis with logistic regression and multivariate analysis with stepwise logistic regression to screen independent predictors for establishing the clinical model.

### Radiomics model development

#### Tumor segmentation and feature extraction

**Fig. 1:**
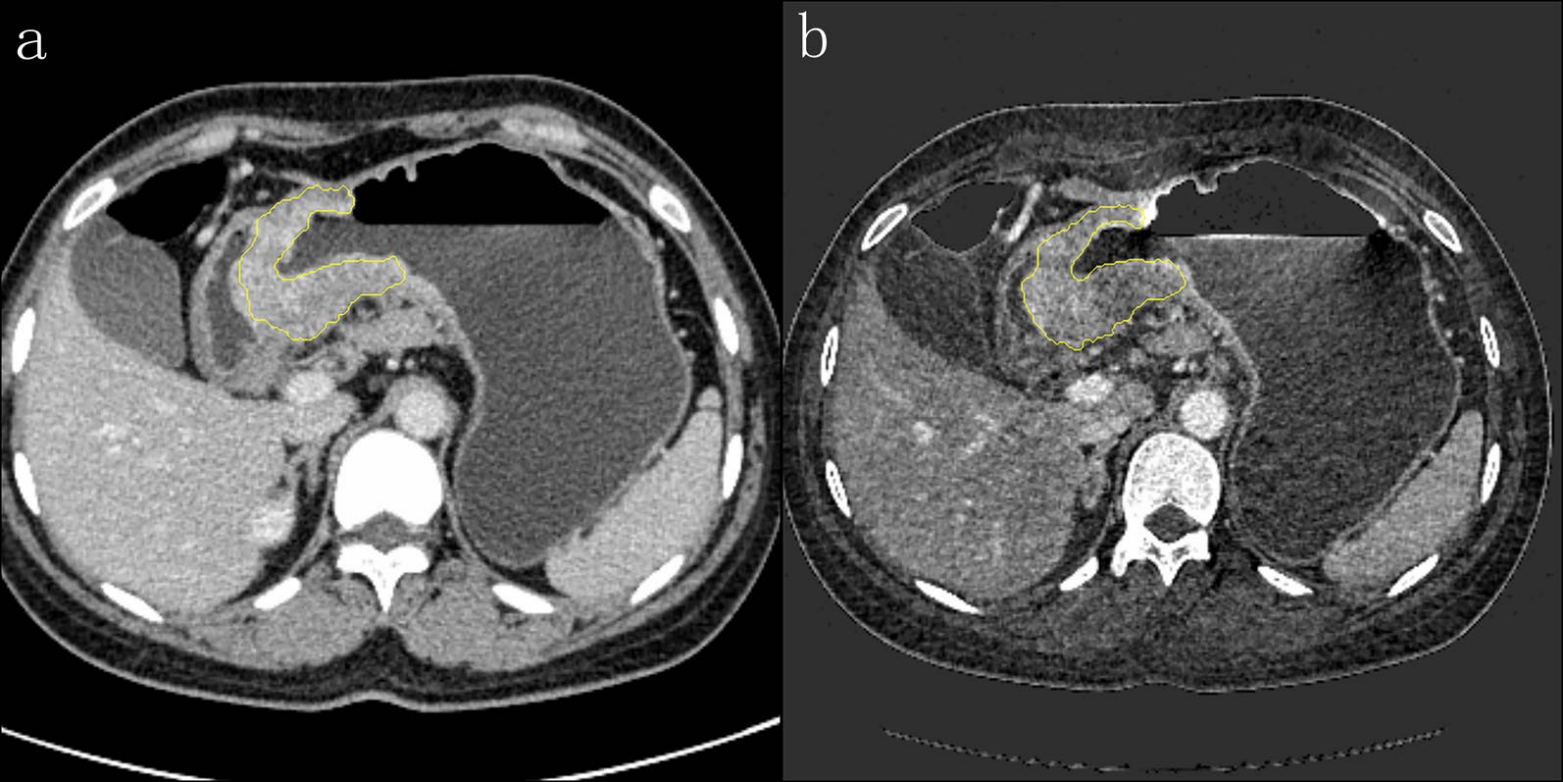
Tumor segmentation method. Axial venous-phase mixed images (**a**) venous-phase iodine map (**b**)

Using the Radiomics software (Frontier, Siemens Healthineers, Forchheim, Germany), observer LM combined the MPR images and outlined layer by layer along the tumor border on the axial venous-phase MIX, avoiding the inclusion of perigastric fat, blood vessels, and gastric contents. The segmentation range was automatically matched to the IM image to generate the same region of interest (ROI). Radiomic features were extracted from an IM of the venous phase and the 120 kVp equivalent MIX, using radiomic software. Detailed settings for radiomic feature extraction in radiomic software (Frontier, Siemens Healthineers, Forchheim, Germany) are provided in Additional file 1. The tumor segmentation method used is shown in Fig. 1.

#### Radiomics feature screening and model development

To reduce feature redundancy and model overfitting, feature screening was performed in the following steps: (1) 1 month after the first tumor segmentation, 50 patients were randomly selected and subdivided by the same observer (L.M.) and another observer (Y.L.) in the same way. The histological characteristics of intragroup and intergroup correlation coefficient (ICC) > 0.8 were retained. (2) We selected predictors using the Boruta algorithm based on a random forest. The radiomics model was constructed with the R package randomForestSRC [22]. Boruta is a recursive feature selection algorithm by disrupting the order of feature variables and calculating their importance in order to select the most important features [23]. There are several methods available for feature selection-based random forest algorithm. The computational efficiency of Boruta is higher for datasets with multiple predictor variables [24]. Using random forests, multiple classification and regression trees are constructed, and the results of each tree are aggregated to produce predictions. As compared to other models, random forest consistently provides high prediction accuracy [25] and isn't prone to overfitting.

Based on the image radiomic features screened by the highest weights in IM and 120 kVp equivalent MIX, we built the IM radiomics model (R-IM model) and MIX radiomics model (R-MIX model), respectively. Likewise, based on the radiomic features screened by the highest weights in IM + MIX, the IM + MIX combined radiomics model (R-COMB model) was established.

**Fig. 2:**
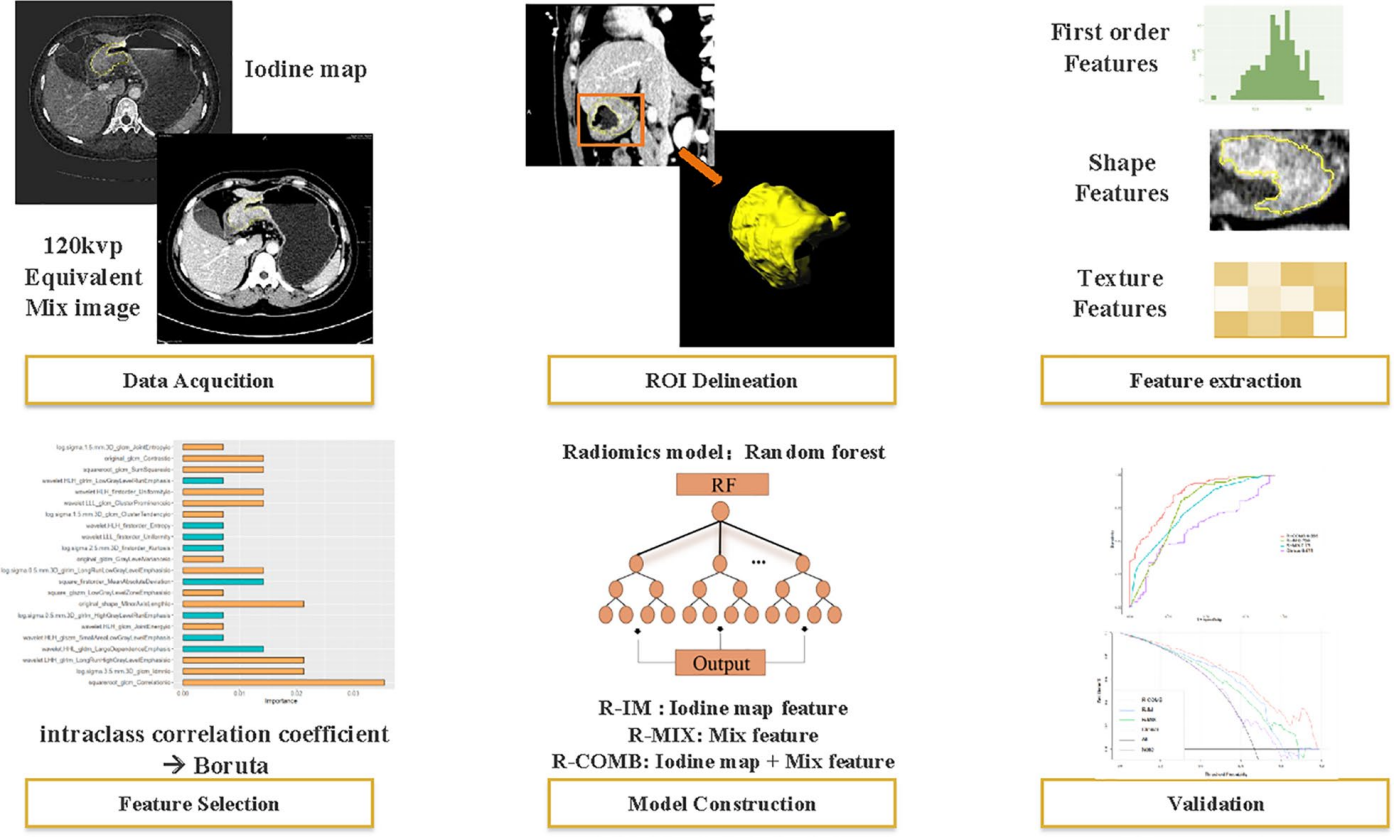
Schematic representation of the radiomics workflow

The radiomics workflow diagram of this study is presented in Fig. 2.

### Statistical methods

R software (version 4.0.3; http://www.Rproject.org) was used for statistical analysis. The Kruskal–Wallis rank sum test was used to compare the continuous variables, whereas the Chi-square or Fisher exact tests were used for categorical variables. Using the receiver operating characteristic (ROC) curve, we evaluated the area under the curve (AUC), accuracy (ACC), sensitivity (SEN), and specificity (SPE) of the model. The performance of each model was compared using the Delong test, and a decision curve analysis (DCA) was done to evaluate the clinical applicability of the model.

## Results

### Comparison of traditional features of patients in the training and testing sets

As shown in Table 1, 240 patients (197 males and 43 females, mean age: 59.8 ± 9.6 years, range 27–80 years) were included in this study. Based on the postoperative pathology to determine the Lauren classification, 80 patients had intestinal-type gastric cancer, 60 cases had a mixed type, and the remaining 100 patients had the diffuse variety.

**Table 1:** Comparison of traditional features of patients in the training and testing sets

Traditional features	Overall (*n* = 240)	Training set (*n* = 168)	Testing set (*n* = 72)	*x*^2^ value	*p* value
Gender				2.611	0.106^a^
Female	43 (17.9%)	35 (20.8%)	8 (11.1%)		
Male	197 (82.1%)	133 (79.2%)	64 (88.9%)		
Age				< 0.001	1.000^a^
< 60 years old	103 (42.9%)	72 (42.9%)	31 (43.1%)		
≥ 60 years old	137 (57.1%)	96 (57.1%)	41 (56.9%)		
Tumor range				1.876	0.171^a^
Single region	176 (73.3%)	128 (76.2%)	48 (66.7%)		
Multi-regions	64 (26.7%)	40 (23.8%)	24 (33.3%)		
Tumor location				0.001	0.977^a^
Proximal	98 (40.8%)	68 (40.5%)	30 (41.7%)		
Distal	142 (59.2%)	100 (59.5%)	42 (58.3%)		
Tumor thickness (cm)	1.37 [1.04; 1.73]	1.37 [1.04; 1.67]	1.37 [1.06; 1.82]	0.229	0.632^b^
Forms of tumor enhancement				3.252	0.071^a^
Uniformity	123 (51.2%)	93 (55.4%)	30 (41.7%)		
Inhomogeneous	117 (48.8%)	75 (44.6%)	42 (58.3%)		
Degree of tumor enhancement				0.048	0.825^a^
Not obviously	66 (27.5%)	45 (26.8%)	21 (29.2%)		
Obviously	174 (72.5%)	123 (73.2%)	51 (70.8%)		
cT staging				0.238	0.626^a^
Non-cT4	60 (25.0%)	44 (26.2%)	16 (22.2%)		
cT4	180 (75.0%)	124 (73.8%)	56 (77.8%)		
cN staging				2.979	0.084^b^
Negative	76 (31.7%)	47 (28.0%)	29 (40.3%)		
Positive	164 (68.3%)	121 (72.0%)	43 (59.7%)		
CEA (μg/L)				0.094	0.758^a^
≤ 5	188 (78.3%)	133 (79.2%)	55 (76.4%)		
> 5	52 (21.7%)	35 (20.8%)	17 (23.6%)		
CA19-9 (kU/L)				1.750	0.186^a^
≤ 27	200 (83.3%)	144 (85.7%)	56 (77.8%)		
> 27	40 (16.7%)	24 (14.3%)	16 (22.2%)		
CA72-4 (kU/L)				0.089	0.765^a^
≤ 6.9	199 (82.9%)	138 (82.1%)	61 (84.7%)		
> 6.9	41 (17.1%)	30 (17.9%)	11 (15.3%)		

Data in parentheses are percentages. Data in the square brackets are quartiles

*CEA* carcinoembryonic antigen, *CA19-9* glycoantigen, *CA72-4* glycoantigen, *cT stage* clinical T stage, *cN stage* clinical N stage

^a^Pearson's Chi-square test

^b^Kruskal–Wallis rank sum test

The enrolled patients were randomly divided in a ratio of 7:3 into a training set (*n* = 168), including 56 cases of intestinal type and 112 cases of non-intestinal type (mixed and diffuse type), and the testing set (*n* = 72), included 24 cases of intestinal type and 48 cases of non-intestinal type. There were no statistically significant differences between the two groups in terms of clinical features (*p* > 0.05). The comparison of baseline information for intestinal and non-intestinal-type patient in the training and test set can be found in Additional file 1: Table S1.

### Clinical features screening and model development

The univariate analysis revealed that gender, tumor extent, tumor morphology, maximum tumor thickness, and cN staging were associated with the Lauren classification (*p* < 0.1). Furthermore, a multivariate by stepwise logistic regression showed that gender (95% 0.194–0.968, OR 0.453; *p* = 0.050) and maximum tumor thickness (95% 1090–2.943, OR 1.748; *p* = 0.027) were independent predictors of the Lauren classification (Table 2). A clinical model was developed based on these two features.

**Table 2:** Univariate and multivariate regression analysis of clinical features

Features	Single factor analysis			Multi-factor analysis		
	OR	95% CI	*p* value	OR	95% CI	*p* value
Gender	0.469	0.201–0.997	0.061	0.453	0.194–0.968	0.050
Tumor range	1.912	1.013–3.778	0.052	–	–	–
Tumor thickness (cm)	1.722	1.073–2.898	0.032	1.748	1.090–2.943	0.027
cN staging	1.616	0.914–2.848	0.096	–	–	–

*cN stage* clinical N stage

### Radiomics model

**Fig. 3:**
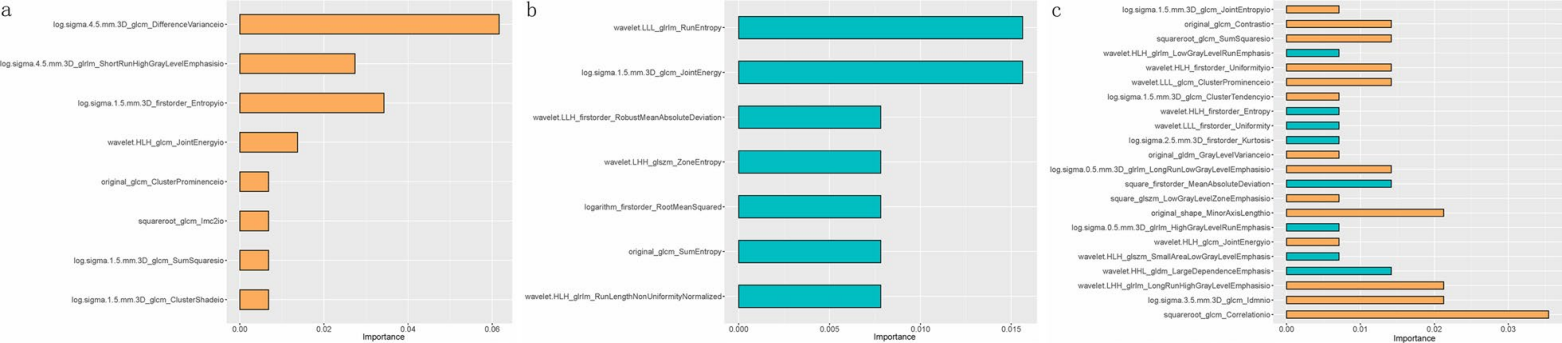
Features importance in three radiomics models. **a** R-IM model; **b** R-MIX model; **c** R-COMB model

A total of 3382 radiomic features (1691 × 2) were extracted from the IM and MIX images of each case; 2482 features with an ICC > 0.80 were further filtered by using Boruta. Eight optimal radiomic features in the IM, including one first-order feature and seven texture features, were selected to build the R-IM model. Seven optimal radiomic features in the MIX images, including two second-order features and five texture features, were selected to build the R-MIX model. The 22 optimal radiomic features in the combined IM and MIX images were selected, including 14 features from the IM—one shape feature, one first-order feature, and 12 texture features, and eight features from the MIX images, including four first-order features and four texture features, were used to build the R-COMB model. The weights occupied by each feature of the three radiomics models are shown in Fig. 3.

### Comparison of the predictive efficacy of the clinical model with the three radiomics models

**Fig. 4:**
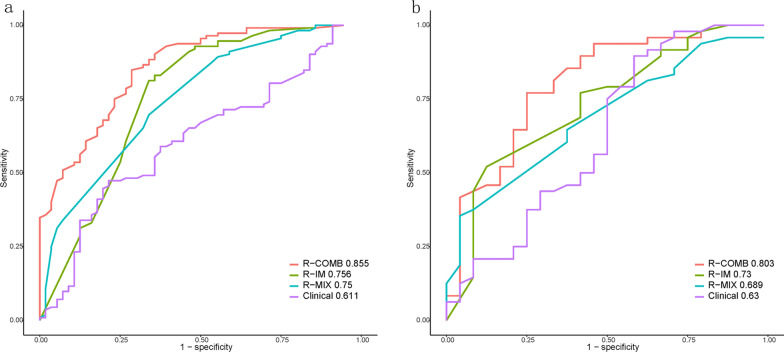
Receiver operating characteristic (ROC) curves of the four models for the training sets (**a**) and testing sets (**b**)

In the training set, the prediction performance of the R-COMB model was superior to the R-IM, the R-MIX, and the clinical models, with AUC values of 0.855, 0.756, 0.75, and 0.611, respectively (*p* < 0.05 each). In the testing set, the prediction performance of the R-COMB model was better than that of the clinical model, with AUC values of 0.803 and 0.630, respectively (*p* < 0.05). However, the differences in the predictive performance of the R-IM and the R-MIX models in the testing set were not statistically significant (*p* > 0.05) Table 3 and Fig. 4.

**Table 3:** Comparison of the prediction performance of different models between the training and testing groups

Models	Training set (*n* = 168)				Testing set (*n* = 72)			
	AUC (95% CI)	Specificity	Sensitivity	Accuracy	AUC (95% CI)	Specificity	Sensitivity	Accuracy
Clinical model	0.611 (0.521–0.700)	0.785	0.473	0.565	0.630 (0.480–0.779)	0.708	0.437	0.527
R-IM model	0.756 (0.670–0.841)	0.642	0.830	0.767	0.730 (0.604–0.850)	0.458	0.791	0.680
R-MIX model	0.750 (0.673–0.826)	0.660	0.696	0.684	0.689 (0.566–0.811)	0.625	0.645	0.638
R-COMB model	0.855 (0.795–0.914)	0.714	0.848	0.803	0.803 (0.690–0.915)	0.750	0.770	0.763

**Fig. 5:**
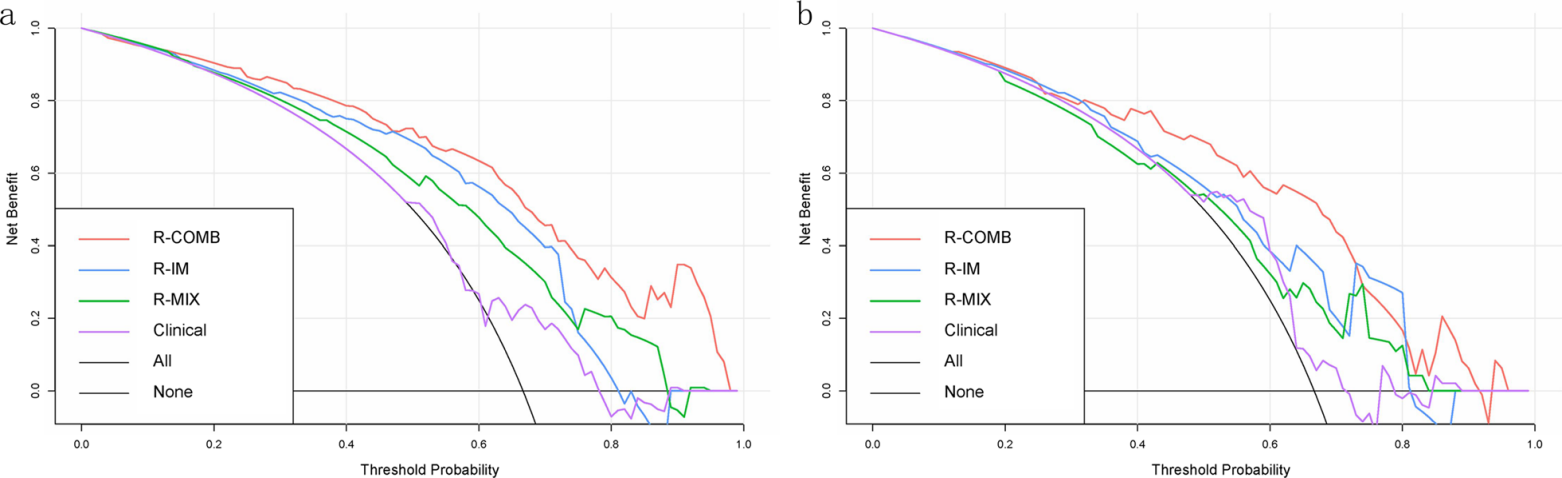
Decision curve analysis (DCA) curves for the four models in the training sets (**a**) and testing sets (**b**)

Furthermore, in the training set, the clinical benefit of the R-COMB model was higher than that of the clinical model, the R-IM model, and the R-MIX model. Whereas in the testing set, the clinical benefit of the R-COMB model was higher than the other three models when the prediction probability was in the range of 0.13–0.25, 0.32–0.72, or 0.81–0.95. These results are presented in Fig. 5.

## Discussion

Based on the DECT venous-phase IM, and MIX images, three radiomics models: R-IM, R-MIX, and R-COMB models were established, respectively. We observed that the prediction performance of each radiomics model in the training set was better than that of the clinical model, and that of the R-COMB model was the best (AUC: 0.855 vs. 0.611). In the testing set as well, the R-COMB model outperformed the clinical model (AUC: 0.803 vs. 0.630). Therefore, we believe that the R-COMB model, based on a combination of DECT venous-phase IM and MIX images, can more accurately predict the Lauren classification of gastric cancer.

The results of univariate logistic regression analysis showed that the traditional clinical features of gender, tumor extent, maximum tumor thickness, and cN staging were correlated with the Lauren classification of gastric cancer. In this group of cases, intestinal type gastric cancer is mostly located in a single region without lymph node metastasis, which is consistent with its biological behavior of weak invasion ability, low heterogeneity, and low sensitivity to lymph node metastasis [3, 5]. Additionally, the results of multivariable logistic regression showed that gender was one of the independent predictors of the Lauren classification of gastric cancer. In our study, the ratio of males to females was 4.58 (197/43). We found that males were more predominant in the intestinal type than in the non-intestinal type (88.7%, 71/80 vs. 78.8%, 126/160), and instead, the number of female patients was higher in the non-intestinal type than in the intestinal type (21.2%, 34/160 vs. 11.3%, 9/80). This result is consistent with the observations of other studies [3, 5]. However, this difference was not statistically significant in this study. This may be related to the limited sample size.

The maximum tumor thickness was another independent predictor of the Lauren classification, with the maximum thickness of intestinal type less than that of non-intestinal types (training set: 1.35 vs. 1.4, test set: 1.13 vs. 1.39). Rossi et al. [26] believe that this is related to the minimal invasiveness of the intestinal type of gastric cancer, infrequent edema of the adjacent gastric wall, and mild fibroproliferative and inflammatory reaction. The predictive efficacy of the clinical model in this study (training sets AUC, 0.611; test sets AUC, 0.630) was comparable to the performance of the clinical model developed in the previous study [27, 28]. This suggests that traditional clinical features have limited predictive value for Lauren classification of gastric cancer.

We used a 3D segmentation tumor for extracting all tumor features [29–31] required to establish radiomics models that can reflect tumor heterogeneity more comprehensively [32]. The prediction performance of our three radiomics models was better than that of the prediction model based on traditional image using 2D segmentation [27]. Among the three radiomics models we developed, the R-COMB model based on IM and 120 kVp equivalent MIX images was the most effective in predicting the Lauren classification of gastric cancer (training sets AUC, 0.855; test sets AUC, 0.802). The results indicate that the predictive ability of the R-COMB model is better than the traditional radiomics model based on CT images reported in reference [33]. The R-COMB model extracted both IM and MIX images in the venous phase, which provided greater quantitative evaluation using radiomics modeling [34–36]. In addition, the IM also contains functional information reflecting tissue perfusion [14, 37], which may be more valuable in model prediction [38]. Notably, the features from the IM accounted for 63.6% (14/22) of the R-COMB model, and the shape, 3D, Long Run High Gray-Level Empha, and square root features of the IM showed significantly higher weights than the radiomics features of MIX images. Therefore, we believe that IM contributes more to the establishment of the R-COMB model. In addition, our R-COMB model has a prediction accuracy of 80.3%, which is higher than preoperative gastroscopy biopsy [13].

Therefore, the R-COMB model based on DECT venous phase IM and 120 kVp equivalent MIX images can serve as a noninvasive and effective new method for predicting preoperative Lauren classification of cancer. This preoperative diagnosis will help in selecting personalized treatment plan and evaluating prognosis.

### Study limitations

First, the retrospective design of the study may have introduced a selection bias. Second, since this was a single-center study with limited sample size and consistent CT scanner, the assessment of model reproducibility with external validation and different CT manufacturers is required in future studies. The script of the model development and validation is available at GitHub (https://github.com/xby947/RF-Model-development.git) to improve the reproducibility of this research. Third, the primary aim of this study is to assess the diagnostic accuracy of dual-energy CT images for Lauren classification. And the further analysis in prognosis of patients with gastric cancer is necessary in following study.

## Supplementary Information

**Additional file 1. Supplementary Table 1:** Comparison of traditional features of patients in the training and testing sets and **Fig 1:** Detailed settings for radiomic feature.

## Data Availability

The datasets generated or analyzed during the study are available from the corresponding author on reasonable request.

## Abbreviations

AJCC: American Joint Committee on Cancer
AUC: Area under the curve
CA19-9: Carbohydrate antigen 199
CA72-4: Carbohydrate antigen 72-4
CEA: Carcinoembryonic antigen
cN: Clinical N
cT: Clinical T
CT: Computed tomography
DCA: Decision curve analysis
DECT: Dual-energy computed tomography
ICC: The intraclass correlation coefficient
IM: Iodine map
MIX: Iodine map and 120 kVp equivalent mixed images
MPR: Multi-planner reformation
R-COMB: Combined IM + MIX image radiomics models
R-IM: IM-radiomics
R-MIX: Mixed radiomics
ROC: Receiver operating characteristic curves
ROI: Region of interest
