# Supplementary material for: Preoperative prediction of Lauren classification in gastric cancer: a radiomics model based on dual-energy CT iodine map
Source: Insights Imaging. 2023 Jul 16;14:125. doi: 10.1186/s13244-023-01477-8 (PMC10350444; doi:10.1186/s13244-023-01477-8)

Preoperative prediction of Lauren classification in gastric cancer: A radiomics model based on dual-energy CT iodine map

ELECTRONIC SUPPLEMENTARY MATERIAL

Supplementary Table 1

Comparison of traditional features of patients in the training and testing sets

	**Training set（n=168）**		**x²-value**	***P*-value**	**Testing set（n=72）**		**x²-value**	***P*-value**
**Traditional Features**	**intestinal （n=56）**	**non-intestinal （n=112）**			**intestinal （n=24）**	**non-intestinal （n=48）**		
**Age**			0.027	0.869[1]			0.856	0.355[1]
<60 years old	23 (41.1%)	49(43.8%)			8(33.3%)	23 (47.9%)		
≥60years old	28 (58.9%)	63(56.2%)			16 (66.7%)	25 (52.1%)		
**Gender**			2.819	0.093[1]			—	0.710[2]
Female	7 (12.5%)	28 (25.0%)			2 (8.33%)	6 (12.5%)		
Male	49(87.5%)	84 (75.0%)			22(91.7%)	42(87.5%)		
**Tumor Range**			0.496	0.481[1]			3.445	0.063[1]
Single Region	45(80.4%)	83 (74.1%)			20(83.3%)	28(58.3%)		
Multi Regions	11(19.6%)	29 (25.9%)			4 (16.7%)	20(41.7%)		
**Tumor Location**			2.597	0.107[1]			0.064	0.800[1]
Proximal	28(50.0%)	40 (35.7%)			9 (37.5%)	21(43.8%)		
Distal	28(50.0%)	72 (64.3%)			15(62.5%)	27(56.2%)		
**Forms of tumor enhancement**			0.027	0.869[1]			3.150	0.076[1]
Uniformity	30(53.6%)	63 (56.2%)			14(58.3%)	16(33.3%)		
Inhomogeneous	26(46.4%)	49 (43.8%)			10(41.7%)	32(66.7%)		
**Degree of tumor enhancement**			0.034	0.853[1]			0	1.000[1]
Not obviously	16(28.6%)	29 (25.9%)			7 (29.2%)	14(29.2%)		
Obviously	40(71.4%)	83 (74.1%)			17(70.8%)	34(70.8%)		
**cT Staging**			0.188	0.664[1]			0.492	0.483[1]
Non-cT4	13(23.2%)	31 (27.7%)			7 (29.2%)	9 (18.8%)		
cT4	43(76.8%)	81 (72.3%)			17(70.8%)	39(81.2%)		
**cN Staging**			1.953	0.162[1]			0.180	0.671[1]

Negative	20(35.7%)	27 (24.1%)			11(45.8%)	18(37.5%)		
Positive	36(64.3%)	85 (75.9%)			13(54.2%)	30(62.5%)		
**CEA (μg/L)**			0.001	1.000[1]			2.781	0.095[1]
≤5	44 (78.6%)	89 (79.5%)			15 (62.5%)	40 (83.3%)		
＞5	12 (21.4%)	23 (20.5%)			9 (37.5%)	8 (16.7%)		
**CA19-9 (kU/L)**			2.679	0.102[1]			0.010	0.920[1]
≤27	52 (92.9%)	92 (82.1%)			18 (75.0%)	38 (79.2%)		
＞27	4 (7.14%)	20 (17.9%)			6 (25.0%)	10 (20.8%)		
**CA72-4（kU/L）**			0.410	0.522[1]			—	1.000[2]
≤6.9	48 (85.7%)	90 (80.4%)			20 (83.3%)	41 (85.4%)		
＞6.9	8 (14.3%)	22 (19.6%)			4 (16.7%)	7 (14.6%)		
**Tumor thickness (cm)**	1.35 [1.04;1.61]	1.40 [1.04;1.75]	1.098	0.294[2]	1.13 [0.70;1.72]	1.39 [1.16;1.86]	3.063	0.080[2]

Data in parentheses are percentages. Data in the square brackets are quartiles. 1Pearson's Chi-square test; 2Kruskal-Walli's rank sum test; CEA: carcinoembryonic antigen, CA19-9: glycoantigen, CA72-4: glycoantigen, cT stage: clinical T stage, cN stage: clinical N stage

Detailed settings for radiomic feature extraction in radiomic software

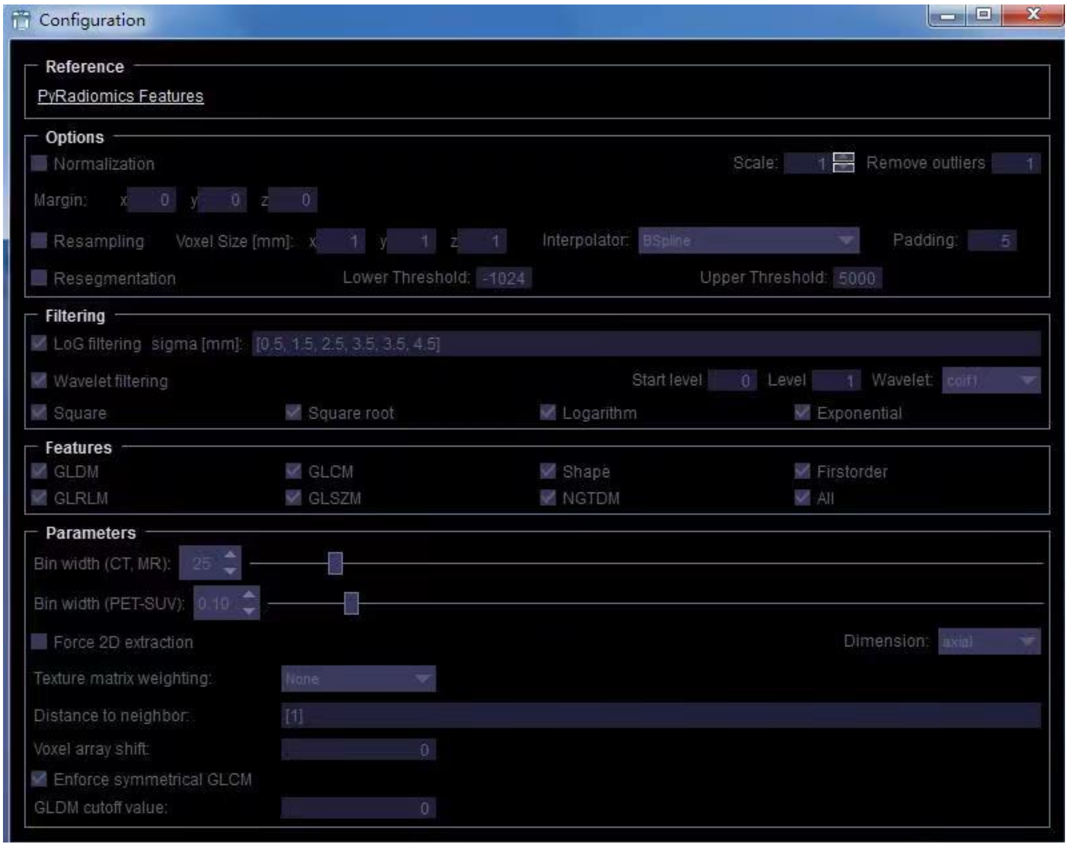

Supplement: Supplementary file 1 — Additional file 1. Supplementary Table 1: Comparison of traditional features of patients in the training and testing sets and Fig 1: Detailed settings for radiomic feature. [file 13244_2023_1477_MOESM1_ESM.pdf]
